# Potential Roles of YAP/TAZ Mechanotransduction in Spaceflight-Induced Liver Dysfunction

**DOI:** 10.3390/ijms24032197

**Published:** 2023-01-22

**Authors:** Wang Li, Xinyu Shu, Xiaoyu Zhang, Ziliang Zhang, Shujin Sun, Ning Li, Mian Long

**Affiliations:** 1 Center for Biomechanics and Bioengineering, Beijing Key Laboratory of Engineered Construction and Mechanobiology and Key Laboratory of Microgravity (National Microgravity Laboratory), Institute of Mechanics, Chinese Academy of Sciences, Beijing 100190, China; 2 School of Engineering Sciences, University of Chinese Academy of Sciences, Beijing 100049, China

**Keywords:** microgravity, liver metabolism, mechanosensing, cytoskeleton, nuclear deformation, phase separation

## Abstract

Microgravity exposure during spaceflight causes the disordered regulation of liver function, presenting a specialized mechano-biological coupling process. While YAP/TAZ serves as a typical mechanosensitive pathway involved in hepatocyte metabolism, it remains unclear whether and how it is correlated with microgravity-induced liver dysfunction. Here, we discussed liver function alterations induced by spaceflight or simulated effects of microgravity on Earth. The roles of YAP/TAZ serving as a potential bridge in connecting liver metabolism with microgravity were specifically summarized. Existing evidence indicated that YAP/TAZ target gene expressions were affected by mechanotransductive pathways and phase separation, reasonably speculating that microgravity might regulate YAP/TAZ activation by disrupting these pathways via cytoskeletal remodeling or nuclear deformation, or disturbing condensates formation via diffusion limit, and then breaking liver homeostasis.

## 1. Introduction

The latest developments in space techniques have allowed increased travel to Earth’s low orbit, space stations or outer space, enabling space travelers to stay in space from hours to years. Notably, the space environment imposes several hazards to these travelers, including microgravity, cosmic radiation, hostile/closed environments, confinement, and distance from earth [1]. Much attention is paid to microgravity, mainly since weightlessness not only leads to the redistribution of body fluid towards the head, but also deprives the human body from gravitational loading [2]. Spending a prolonged amount of time in space brings increased risks of reduced cardiovascular capacity, impaired immune system, declined bone density, disordered neurological function, and atrophied muscle [3,4,5]. Similar to other tissues and organs, the liver, the largest visceral organ involved in detoxication and metabolism to maintain body homeostasis, also experiences remarkable gravity changes. Comparisons between inflight specimens and ground-based controls presented the largest number of differentially expressed genes and proteins in liver among ten types of mouse tissues including muscles, eyes, and kidneys [6]. As a guard against drug-induced injury in the body, the liver has a significant impact on the pharmacokinetic and pharmacodynamic properties of medications that are taken during spaceflight [7,8]. In addition, microgravity exposure dysregulates liver functions, resulting in apoptosis and oxidative stress-associated liver injury and inflammation, compromised carbohydrate metabolism, alterations in hepatic xenobiotic biotransformation machinery, and lipids deposition [9]. Evidently, these dysfunctions are correlated with microgravity-induced alterations of parenchymal and non-parenchymal cells inside the liver. How these hepatic cells sense, transmit and respond to microgravity through mechanotransductive pathways is currently poorly understood.

Existing evidence in space or on the ground provides limited cues along this line. To date, only one report discusses the cellular response of hepatocyte-like cells under real space microgravity. After being cultured in space for 16 days, a PICM-19 pig liver stem cell line tended to differentiate into hepatocytes normally with a well-formed biliary canaliculi structure [10]. Inducible cytochrome P450 (CYP) activities, urea secretion, and expressions of liver-specific genes were also maintained. Noteworthily, no significant discrepancy was discovered between flight specimens and ground controls, except for minor changes in gene expression, which might result from the small sample size. In contrast, several ground-based studies elaborated on the simulated effects of microgravity on hepatocyte metabolism using a typical rotation culture. While diversified results still existed, most outcomes presented improved hepatocyte functions, such as albumin production and drug metabolism. However, the underlying mechano-biological coupling mechanisms are still far from being elucidated [9,11,12].

At the molecular level, multiple mechanosensitive proteins may serve as the candidates to sense and respond to microgravity. While it is still quite tough to define the membrane-bound mechanosensor on mammalian cells, it is relatively straightforward to elucidate the intracellular mechanosignaling molecules. One of the potential regulators may be paralogous transcription coactivators yes-associated protein/transcriptional coactivator with PDZ-binding motif (YAP/TAZ), two key mechanosensitive proteins involved in regulating cell differentiation, proliferation, apoptosis, and metabolism [13]. The protein YAP/TAZ shuttles between the nucleus and the cytoplasm and subsequently initiates or prevents various transcriptional responses once perceiving mechanical cues, including microgravity in particular [14,15]. Specifically, activated YAP/TAZ pathways can promote glycolysis, lipogenesis, glutaminolysis, and amino acid transport [16]. These findings suggest YAP/TAZ may function as an emerging bridge connecting microgravity and liver dysfunction.

In this review, the effects of spaceflight and ground-based simulated weightlessness on liver functions, especially on metabolic dysfunctions, were summarized. Contributions of YAP/TAZ to liver functions and potential responses of YAP/TAZ to microgravity were elaborated on. The roles of cytoskeleton reorganization, nuclear deformation, or phase separation in YAP/TAZ activation were discussed. Finally, this mechanotransductive pathway was proposed to be likely engaged in spaceflight-induced liver dysfunctions.

## 2. Microgravity Induces Liver Dysfunction

An increasing amount of evidence indicates the potential liver dysfunction induced by microgravity. Existing data seem quite diverse, spanning from inflight to ground-based studies, from organism to cell level, and one species to another. Here, we provide a brief update on the progress made in this area, specifically regarding biological functions and cellular phenotypes.

### 2.1. Inflight Studies

The liver is responsible for various physiological functions in the human body, such as plasma protein production, bile secretion, the metabolic process, and immune defense. Microgravity-induced liver dysfunction is a challenge, especially in long-duration space missions (Table 1). Japanese quail on board the orbital station Mir for 5 days displayed extensive deposits of lipid droplets in almost all hepatocytes of all the flight chicks, and the overall amount of lipid droplets was nearly 5-fold higher than ground control chicks [17]. Meanwhile, the accumulation of lipids in the liver led to a depletion of adipocytes in the bone marrow of these flight chicks. Abnormal lipid accumulation measured by oil red O staining was also visualized in all space-flown mice liver [18]. RNA-sequencing data indicated that responsive pathways were related to the upregulation of lipid and fatty acid metabolism and processing, lipid catabolic processing, and lipid localization, as supported in a quantitative proteomic analysis detailing the changes in lipid-related proteins. Both gene and protein data identified the activation of lipotoxic pathways in the liver. Further exploration showed that retinoids were lost from lipid droplets during spaceflight. This might be associated with the activation of the peroxisome proliferator-activated receptor (PPAR) alpha-mediated pathway and hepatic stellate cells (HSCs), which led to the remodeling of the extracellular matrix (ECM) and the early signs of liver injury [19]. In comparison with the on-orbit group, the members of PPAR pathway decreased their protein expressions after landing and re-adaption to normal gravity for 7 days on Earth, indicating the diminished liver lipotoxicity [20]. In contrast, proteins related to amino acid metabolism increased after re-adaption, implying a high rate of gluconeogenesis. These findings were supported by elevated glycogen storage in rat liver flown on Cosmos 2044, accompanied by significantly increased enzyme activities of tyrosine aminotransferase and tryptophan oxygenase for amino acid metabolism [21]. Consistently, weightless flight led to more than twice the amount of glycogen contents and decreased the activity of glycogen phosphorylase (an enzyme related to glycogen breakdown) in rat liver, despite the unchanged activity of glycogen synthetase [22]. Functional pathway analysis of gene expression suggested that the generation and sequestration of glucose were upregulated in space-flown mouse liver, which was in accordance with a downregulated glycolysis process, declined glycogen phosphorylase expression, and augmented glycogen synthetase level, but was contradictory with a profound loss of glycogen storage [23]. Thus, these cues implied that lipid deposition and glycogen synthesis were augmented by microgravity.

Besides glucolipid metabolism, liver plays a central role in converting xenobiotics into metabolites through three major pathways: phase I, phase II, and phase III [24]. Rats flown for 14 days showed decreased phase I enzyme expression of CYP, while the phase II enzyme level of glutathione-S-transferase was not altered [25]. On the contrary, mice exposed to 30 days of spaceflight displayed increased productions of CYP2C29, CYP1A2, and CYP2E1 [26]. Within 7 days after landing, the former two CYP subtype contents returned to the baseline level, whereas the elevated CYP2E1 expression still maintained high level. The amount of CYP4A1 was also increased in rats flown for 12 days, but other CYP genes examined did not change [27]. These observations suggest the diverse effects of microgravity on CYP metabolism.

**Table 1:** Effects of spaceflight on liver function.

Object	Method (Space Mission)	Duration	Outcome	Refs
PICM-19 pig liver stem cells	Space shuttle (STS-126)	16 days	CYP450 N.S. Urea secretion N.S. Liver-specific genes N.S.	[10]
Japanese quail liver	Orbital station Mir	5 days	Number of lipid droplets ↑	[17]
Mouse liver	ISS (RR-1, RR-3) and Space shuttle (STS-135)	13.5~42 days	Lipid deposition ↑ Lipid metabolism ↑ Lipotoxic pathways ↑	[18]
Mouse liver	Space shuttle (STS-135)	13.5 days	Lipid droplets ↑ HSCs activation ↑ ECM remodeling ↑ PPAR pathways ↑ Retinol storage in HSCs lipid droplets ↓	[19]
Rat liver	Satellite (Cosmos 2044)	14 days	Glycogen storage ↑ Tyrosine aminotransferase ↑ Tryptophan oxygenase ↑ Cholesterol and sphingolipids ↓ δ-aminolevulinic acid synthase ↓ CYP450 N.S.	[21]
Rat liver	Satellite (Cosmos 936)	18.5 days	Glycogen storage ↑ Palmitoyl CoA desaturase ↑ Glycogen phosphorylase ↓ α-glycerol phosphate acyltransferase ↓ Diglyceride acyltransferase ↓ Aconitase ↓ 6-phosphogluconate dehydrogenase ↓	[22]
Mouse liver	Space shuttle (STS-135)	13.5 days	Fatty acid oxidation ↑ Viral infection defense ↑ Phagocytosis ↑ CD8^+^ T cells activation ↓ Glycolysis ↓ Glycogen storage ↓	[23]
Rat liver	Space shuttle (SLS-2)	14 days	CYP450 ↓	[25]
Mouse liver	Satellite (Bion-M1)	30 days	CYP1A2, 2C29, 2E1 ↑	[26]
Mouse liver	Space shuttle (STS-108)	12 days	CYP4A1 ↑ T cells activation ↑ CDK inhibitor 1A ↑ Apoptosis ↑ Cell death ↑	[27]
Mouse liver	ISS (Kibo)	35 days	Hepatic cells proliferation in offspring ↑	[28]
Rat liver	Space shuttle (SLS-1)	9 days	Glycogen storage ↑ Lipid deposition ↑ CYP4A1 ↑ Crip ↑ HSP90 ↓ p53 ↓ Glutathione-S-transferase N.S.	[25,29]
Mouse liver	Space shuttle (STS-135)	13.5 days	Reactive oxygen species ↑ Autophagy ↑ Proteasome ↑ Lipid deposition ↑ Hepatocyte senescence ↑ Glutathione levels ↓ NFE2L2-mediated pathway ↓	[30]
Human blood	ISS	180 days	Total cholesterol ↑ Low-density lipoprotein ↑ High-density lipoprotein ↓	[6]

N.S., not significant. ↑, upregulation. ↓, downregulation.

Moreover, hepatocyte proliferation tended to be inhibited after spaceflight, since cyclin-dependent kinase (CDK) inhibitor 1A, an inhibitor of DNA replication, and those genes related to apoptosis and cell death were particularly overexpressed [27]. However, male mice subjected to 35 days spaceflight exhibited alterations in DNA binding of transcription factor ATF7 and small RNA expression in spermatozoa, promoting hepatic DNA replication in offspring with intergenerational effects [28]. Besides cell proliferation, hepatic injury in response to microgravity was taken into investigation. In the rat flown for 9 days, the expressions of stress sensors such as cold-inducible RNA binding protein (Crip), heat shock protein 70 (Hsp70), Hsp90, and the p53 tumor suppressor gene were determined [29]. Compared with ground control, Crip expression was significantly upregulated while Hsp90 and p53 expressions were downregulated in the flight group, suggesting that the liver was vulnerable in response to microgravity stimuli. This speculation was further supported by the findings that the increased oxidative stress due to elevated reactive oxygen species and the downregulated nuclear factor (erythroid-derived 2)-like 2 (NFE2L2)-mediated signaling impaired liver protection from oxidative attack after exposure to the space environment, and finally gave rise to the activation of hepatocyte senescence [30]. Combined with upregulated autophagy and the ubiquitin-proteasome, degradation programs were also induced in the liver. In addition to increased oxidative stress, microarray analysis revealed immune function in liver was dysregulated by spaceflight [23]. For example, the gene expressions related to viral infection and phagocytosis were increased, despite a surprising downregulation in the functional activation of CD8^+^ T lymphocytes. It was also controversial that genes related to T cell activation were upregulated in liver tissue from mice flown in space [27], implying the complexity of hepatic immune responses in space.

Recent studies have started to extend existing knowledge on liver function in rodents to the human body. Astronauts exhibited increased levels of total cholesterol and low-density lipoprotein but decreased levels of high-density lipoprotein in the blood after travelling in space for 180 days, all of which reverted to baseline levels after 30 days postflight [6]. Consistent with enhanced gluconeogenesis observed in mouse liver [20], increased plasma glucose concentration was seen in astronauts after spaceflight [31].

Thus, it is reasonably concluded that spaceflight upregulates lipid accumulation, lipid and fatty acid metabolism, amino acid metabolism, glucose production, and gluconeogenesis, while it downregulates glycolysis but induces controversial variations in glycogen storage and CYP450 expressions. These conflicting observations may result from the discrepancy presented in animal species, sex, age and feeding conditions, as well as flight duration. Of note, liver functional evaluation carried out on the human body is usually limited to blood or urine samples. In animal experiments, it is difficult to expand the sample size and sort out the impacts of specific cell types accounting for liver dysfunctions. To further decipher the mechanisms of liver function dysregulated by spaceflight, experimental explorations at the cellular level are in great demand, since relevant studies are still lacking, as introduced above.

### 2.2. Ground-Based Studies

It is difficult to perform all the experiments in space due to the high costs, complex equipment, and long preparatory period. Alternatively, several ground-based models have been developed to investigate the simulated effects of weightlessness on liver functions (Table 2), including tail suspension for rodents and head-down bed rest for human beings. Rats exposed to tail suspension showed reduced levels of serum glucose and hepatic glycogen but elevated levels of alanine aminotransferase (ALT) and aspartate aminotransferase (AST), along with enhanced expressions of Bax, Bcl-2, and active caspase-3, indicating liver injury and cells apoptosis [32,33]. Hepatocyte proliferation was negatively regulated by miR-223 in rats under tail suspension [33]. In the human body, head-down bed rest studies showed that short-term duration (12 h) led to a decline in metabolic and detoxified capacities because of reduced blood flow passing through the liver [34], while long-term duration (85 days) caused enlarged cross-sectional area and enhanced flow volume in the portal vein [35]. It was also indicated that five-day bed rest induced non-inflammatory shedding of L-selectin into plasma samples from granulocytes [36], serving as a potential regulator in localized hepatic inflammation. Evidently, these in vivo approaches provide crucial cues for understanding the roles of these simulating effects in liver function, although a full understanding is yet to be obtained due to the complexity of the field.

In addition to the above studies, at the individual organism level, several clinostat systems were applied to simulate microgravity effects, including a rotating wall vessel (RWV), also known as a rotary cell culture system (RCCS), a random positioning machine (RPM), and other two-dimensional (2D) or 3D clinostats (Table 2) [4,37]. Hepatocellular carcinoma (HCC) cell lines or primary hepatocytes cultured in RWV had a cellular morphology reminiscent of in vivo hepatic tissue; that is, a well-formed bile canaliculus-like structure, intact microvilli, and clearly presented tight junctions [38,39,40]. Albumin synthesis, α1 antitrypsin expression, ammonium consumption, and glycogen storage were enhanced, although multiple drug-metabolizing enzyme activities were controversial [41,42,43,44,45]. Lipid metabolism might not be fostered in RWV culture because those lipid transporter activities, encoded by three genes of apolipoprotein A1 (APOA1), APOA2, and APOB, were significantly downregulated [46]. Hepatocyte proliferation showed opposite results under varied conditions, exemplifying that cell proliferation was promoted for HepG2 cells rather than mouse primary hepatocytes cultured in RWV [41,47], but inhibited for CCL-13 or HepG2 cells cultured in 3D clinostat [48,49]. Specifically for the latter, cell cycle was arrested by downregulated protein expressions of cyclin A1, cyclin A2, cyclin D1, and CDK6, and apoptotic signaling was enhanced by the activation of caspase-3. Additionally, a downregulated gene, secreted protein acidic and rich in cysteine (SPARC), was screened out from RPM assay, serving as a potential microgravity sensor in liver [50]. Thus, these findings provide cues for understanding the regulations of hepatocyte functions by simulating the effects of microgravity.

**Table 2:** Effects of ground-based simulated weightlessness on liver function.

Object	Method	Condition	Outcome	Refs
Rat liver	Tail suspension	2 months	AST, ALT ↑ Apoptosis ↑ Serum glucose ↓ Glycogen storage ↓	[32]
Rat liver	Tail suspension	14~42 days	AST, ALT ↑ Alkaline phosphatase ↓ Hepatocyte proliferation ↓	[33]
Human liver	Head-down	−15°, 12 h	Portal vein blood flow ↓	[34]
Human liver	Head-down	−6°, 85 days	Portal vein blood flow ↑ Portal vein cross-section area ↑	[35]
Porcine primary hepatocyte	RWV	10~15 rpm, 12 days	Albumin ↑ Hepatocyte polarity ↑ α5 integrin ↑	[39]
HepG2 cells	RWV	20~30 rpm, 1~15 days	Lactate dehydrogenase ↑ Alpha-fetoprotein ↑ CD29, CD44, CD54 ↑ E-cadherin ↓ Glucose consumption ↓	[40]
HepG2 cells	RWV	1~10 days	Albumin ↑ α1 antitrypsin ↑ Proliferation ↑ CYP1A1, 1A2 ↓	[41]
Mouse fetal liver cells	RWV	15~18.5 rpm, 5~10 days	Albumin ↑ α1 antitrypsin ↑ Glucose-6-phosphatase ↑ Tryptophan-2,3-dioxygenase ↑ Asialoglycoprotein receptor ↑ Ornithine transcarbamylase ↑ Ammonia elimination ↑ CYP3A ↑	[43]
HepG2 cells	RWV	16~20 rpm, 6 h~7 days	Albumin ↑ CYP450 ↑ ECM ↓ APOA1, APOA2, APOB ↓	[44,46]
Mouse primary hepatocytes	RWV	16 rpm, 4 h~3 days	Albumin ↑ CYP1A1 ↑ Metabolic genes ↑ Mesenchymal genes ↓ Cytoskeletal genes ↓ Proliferation ↓	[47]
CCL-13 cells	3D clinostat	72 h	Proliferation ↓ α-tubulin 3, β-actin ↓	[48]
HepG2 cells	3D clinostat	0~3 days	Apoptosis ↑ Autophagy ↑	[49]
HepG2/C3A cells	Clinostat	22~25 days	Drug metabolism ↑	[51]

↑, upregulation. ↓, downregulation.

Collectively, the simulated effects of microgravity on physiological consequences yield potential liver injury and loss of metabolic abilities on the ground. Nevertheless, almost all the findings in various clinostats display well-differentiated hepatocyte morphology and matured liver-specific functions, which might account for beneficial 3D spheroid growth or better mass transfer occurrence in clinostats culture [51,52,53]. Although these models applied for simulating microgravity effects have been used for a long time, the results so obtained still need to be interpreted carefully, since remarkable differences indeed exist between ground-based models and real microgravity in space, based on underlying physical rationale. The conclusions from these ground-based studies on the simulated effects of microgravity should be further verified in space.

## 3. YAP/TAZ May Bridge Microgravity and Liver Dysfunction

Whether or not the data in liver functions are derived from infight or ground-based studies, or what types of observations are presented at the organism or cellular level, it is still critical to map out the potential gravity-sensitive signaling pathways from the above functional or phenotypic cues. Here, we focused on a typical mechanosensitive protein, YAP/TAZ, mainly based on the below understanding.

### 3.1. YAP/TAZ Is Essential for Liver Metabolism

**Figure 1:**
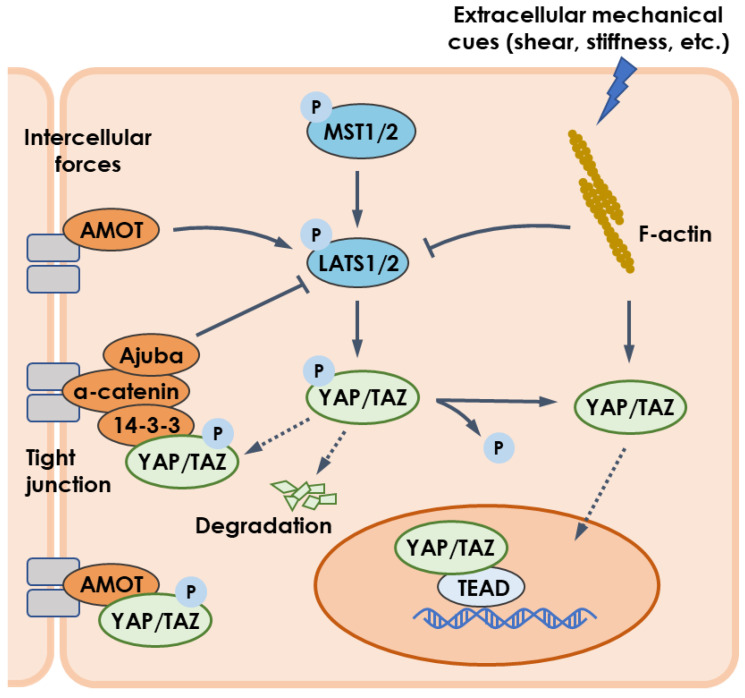
YAP/TAZ pathway regulated by mechanical cues. Intercellular forces enable LATS1/2 to be upregulated by AMOT but inhibited by Ajuba. Mechanical cues such as flow shear and substrate stiffness regulate actin cytoskeleton to activate YAP/TAZ pathway via downregulating LAST1/2 or upregulating YAP/TAZ. Besides, YAP/TAZ is sequestrated in cytoplasm by physical interaction with AMOT. Arrows, blunt ends, and dashed lines indicate activation, inhibition, and translocation, respectively.

Typically, YAP and TAZ are regarded as the effectors of the Hippo cascade of kinases, the core components of which comprise the mammalian STE20-like protein 1/2 (MST1/2) kinase and large tumor suppressor 1/2 (LATS1/2) kinase (Figure 1) [54]. Activated MST1/2 induces the phosphorylation and activation of LATS1/2. In turn, LATS1/2 promotes the phosphorylation of YAP/TAZ, resulting in cytoplasmic retention by binding to 14-3-3 protein and proteasomal degradation [55,56,57]. Conversely, those YAP/TAZ proteins not phosphorylated can enter the nucleus and initiate gene transcriptions, predominantly depending on their interaction with the TEA domain (TEAD) family [58].

**Figure 2:**
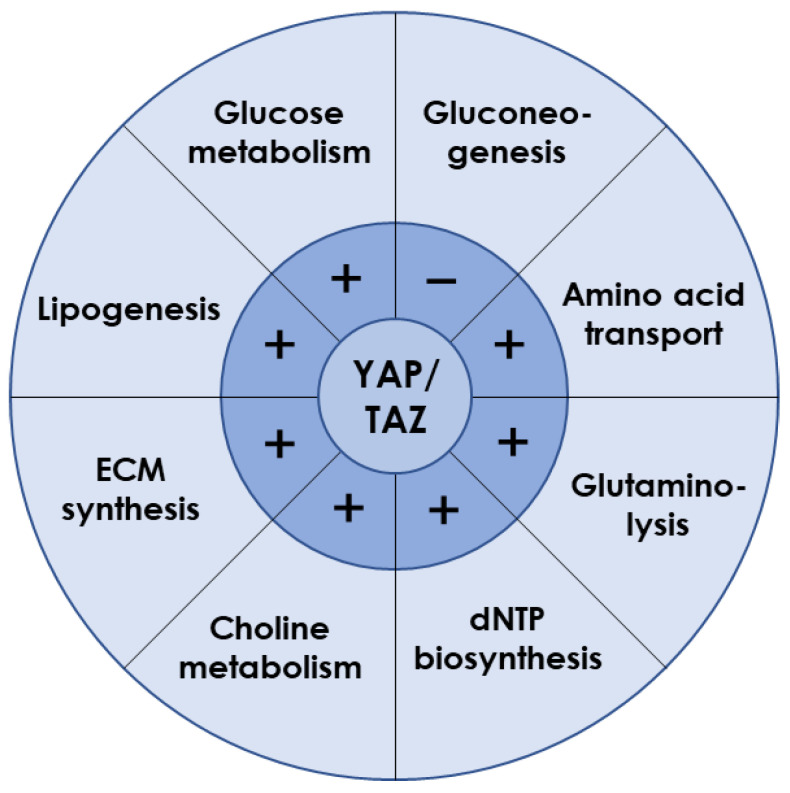
Liver metabolism regulated by YAP/TAZ. YAP/TAZ is required for the expression of key transporters or enzymes involved in various metabolic processes. Those plus and minus signs indicate the positive and negative regulation, respectively.

Although the main issues of YAP/TAZ signaling concentrate on cell proliferation and differentiation, increasing evidence indicates their key roles in hepatocyte metabolism (Figure 2) [16,59]. For example, YAP knockdown reduced lipid droplets deposition in liver tumor cells induced by anti-PD-1 treatment, suggesting YAP promoted lipid uptake and synthesis [60]. This is in line with a study using the dexamethasone-induced hepatomegaly model, where the pregnane X receptor/YAP pathway was activated together with enhanced uptake of fatty acids and suppressed lipolysis and fatty acid β-oxidation [61]. Furthermore, overexpressed YAP in HCC cells promoted lipid peroxides production by transcriptionally upregulating the arachidonate lipoxygenase 3, which sensitized HCC cells to ferroptosis [62]. Given that YAP inhibition in mammary epithelial cells or prostate cancer cells reduced free fatty acid contents but increased triglyceride and cholesterol levels within cells [63,64], YAP-mediated lipid metabolism may vary with cell types.

Glucose metabolism is a major energy source for supporting cellular activities in liver. As the exclusive pathway for initiating glucose metabolism, glycolysis was found to be prevalent in energy-consuming tumor cells, along with frequent YAP nuclear translocation [65]. Indeed, YAP activation increased ATP content, glucose consumption, lactic acid production, extracellular acidification rate, and glycolysis-related enzymes expression [66,67,68], indicating the accelerated glycolytic rate. Hexokinase 2, lactate dehydrogenase A, glucose transporter 1 (GLUT1), and 6-phosphofructo-2-kinase/fructose-2,6-biphosphatase 3/4 (PFKFB3/4) contribute to glucose uptake [69,70]. GLUT1 was identified as the direct transcriptional target of YAP/TEAD1 to promote the glycolysis process [66,71,72]. In addition, the hypoxic microenvironment also helped YAP to be liberated from cytoplasm and stabilized in the nucleus [73,74,75,76]. Glycolysis was enhanced when YAP bound the promoters of pyruvate kinase M2 or PFKFB3 with the assistance of hypoxia-inducible factor 1α or TEAD1, respectively [77,78]. When blood glucose is too low to meet body requirements, gluconeogenesis is initiated by glucagon. In a capping actin protein of a muscle Z-line (CAPZ) liver-specific knockout mouse, YAP hyperactivation was observed to be associated with decreased gene expressions of glucose-6-phosphatase, phosphoenolpyruvate carboxylase 1, and fructose-1,6-bisphosphatase 1, serving as the key genes required for gluconeogenesis [79]. Loss-and-gain functional experiments in mouse primary hepatocytes demonstrated that active YAP inhibited gluconeogenic gene expression induced by glucagon or dexamethasone treatment, through preventing peroxisome proliferator–activated receptor-gamma coactivator 1 from binding to the promoters of gluconeogenic targets [80]. Overall, YAP plays a key role in promoting glucose uptake and consumption while inhibiting gluconeogenesis and, hence, is essential for maintaining glucose homeostasis in the whole organism [81,82].

YAP/TAZ is also required for amino acid metabolism in liver. For instance, the serine/glycine-producing enzymes were increased by YAP overexpression [83]. Activated YAP/TAZ upregulated the amino acid transporters of solute carrier 38A1 (SLC38A1), SLC7A5, and SLC3A2 to supply sources of essential amino acid synthesis [84,85]. Glutamine is the most abundant nonessential amino acid and can be used as the complement of intermediate products in the tricarboxylic acid after deamination by glutaminase and subsequent conversion into α-ketoglutarate [86]. Knockdown of YAP or TAZ could reduce intracellular glutamate concentrations with different mechanisms, where YAP deletion reduced expression of SLC1A5 but not glutaminase, while TAZ deletion decreased both SLC1A5 and glutaminase expression. Experiments in zebrafish indicated that YAP reprogramed glutamine metabolism by upregulating the expression and activity of glutamine synthetase to drive nucleotide biosynthesis [87].

Furthermore, YAP/TAZ participates in other metabolic processes in liver. It directly regulated the expression of key enzymes involved in deoxynucleoside triphosphates (dNTP) biosynthesis required for DNA replication and dNTP precursor pools maintenance, enabling cells to resist chemotherapeutics targeting dNTP synthesis and to avoid oncogene-induced senescence phenotype [88]. In addition, activated YAP promoted choline metabolism by inducing expression of those genes involved in apical extrusion during cell competition [89]. Moreover, YAP overexpression promoted the synthesis of ECM components such as aggrecan, type II collagen, and sulfated glycosaminoglycan [90].

Evidently, YAP/TAZ is known to positively regulate lipogenesis, glucose metabolism, amino acid metabolism, glutaminolysis, dNTP synthesis, choline metabolism, and ECM synthesis, but to negatively regulate gluconeogenesis (Figure 2). Interestingly, liver metabolism after YAP/TAZ inhibition is consistent with those functional changes induced by microgravity. Therefore, it reasonably anticipates that YAP/TAZ may act as a key modulator when it reacts to microgravity stimuli.

### 3.2. Microgravity Regulates YAP/TAZ Activation

Increasing knowledge suggests that YAP/TAZ can respond to microgravity, although such responses have been poorly understood in hepatocytes. When cardiovascular progenitor cells (CPCs) were cultured on the International Space Station or with 2D clinostat on the ground, both spaceflight and simulated effects of microgravity were able to induce gene expressions of YAP and its target, superoxide dismutase 2, a marker of cell survival [15]. However, this finding was limited to CPCs derived from adult humans, because the YAP gene was downregulated by spaceflight in neonatal CPCs [91]. In human colorectal cancer cell HCT116, simulated effects of microgravity using RWV promoted YAP nuclear localization, which was correlated with stemness marker expressions of octamer-binding transcription factor 4 (Oct4), SRY-box 2 (Sox2), homeobox protein nanog (Nanog), and NK2 homeobox 5 [92]. On the contrary, YAP nuclear entry was impaired in glioblastoma cells or mesenchymal stem cells (MSCs) when cells were cultured within RPM or clinostat, respectively [93,94]. By utilizing a 2D clinostat designed in our own lab, the activation and expression of TAZ were hindered by depolymerizing F-actin, stemmed from the simulated effects of microgravity, which accounted for the inhibited osteogenic differentiation of MSCs [95,96]. Furthermore, human microvascular endothelial cells subjected to hypergravity by centrifuge (4 g for 15 min or 20 g for 15 min~6 h) presented a dose-dependent increase in phosphorylated focal adhesion kinase, YAP, and myosin fibers, as well as improved tube formation, indicating a seemingly opposite response to microgravity in the same cell type [97,98]. Taken together, the YAP/TAZ pathway is positively or negatively regulated by microgravity stimuli in an experimental condition- or cell type-specific manner. It is yet difficult to draw an unambiguous conclusion from those studies since YAP/TAZ activation has not been measured in different cell types upon identical microgravity exposure or in the identical cell type under various microgravity loading modes. Nevertheless, space microgravity is assumed to be sensed by the YAP/TAZ molecule at the cellular level, although the elaborative perception mechanism remains obscure. Further exploration of whether YAP/TAZ is affected and how it is exactly inhibited by microgravity loading in hepatocytes, finally inducing liver dysfunction, is still needed.

## 4. YAP/TAZ Pathway Could Be Specialized in Microgravity-Induced Liver Dysfunction

It is speculated that YAP/TAZ might be involved in microgravity-dysregulated liver functions, based on the above discussions. In addition to those biological and biomedical viewpoints, here, we just focused on two possible ways proposed for how YAP/TAZ signaling responds to microgravity, one from mechanotransductive pathways and another from phase separation.

### 4.1. Mechanotransduction and YAP/TAZ Pathway

Growing evidence suggests that mechanical stimuli can regulate the cellular distribution of YAP/YAZ [99]. For example, YAP/YAZ was localized in the cytoplasm of cells attached to a confined adhesive area or to a soft ECM matrix [100,101], but transferred to the nucleus for cells experiencing laminar flow or mechanical stretch [102,103]. This regulation depends on mechanotransduction, in which cells sense physical cues from intercellular forces via Ajuba or Angiomotin [104,105,106] or from extracellular stimulation (e.g., blood flow) via actin cytoskeleton [107], and then mount adaptive responses of YAP/TAZ (Figure 1). It is still unknown whether and how this mechanism is suitable for the YAP/TAZ pathway regulated by microgravity, a special mechanical loading mode.

Mechanical loads containing pulling, compressing, and shearing are usually perceived by cells through cell-ECM focal adhesion or cell-cell interconnection, and then transmit toward the nucleus through the cytoskeleton. Notably, these mechanosensory proteins are likely implicated in YAP and TAZ regulation. For example, either disruption of F-actin or inhibition of myosin contraction abolished YAP/TAZ activation [107,108], suggesting that the integrity and contraction ability of actin cytoskeleton were required for YAP/TAZ mechanotransduction. Moreover, loss of F-actin capping or severing proteins, such as CAPZ, cofilin, and gelsolin [79,109], led to YAP/TAZ hyperactivation, indicating that reorganization of actin cytoskeleton favored to accomplish YAP/TAZ mechanotransduction. In space, osteoblasts showed disrupted cytoskeleton with disassembled F-actin fibers, shorter and wavier microtubules, as well as decreased α-tubulin mRNA after spaceflight [110,111]. For endothelial cells aboard the SJ-10 recoverable scientific satellite for 10 days, the vimentin, an intermediate filament protein, was increased in addition to the deceased amounts of β-actin and α-tubulin [112,113]. The distribution of cytoskeletal components was rearranged in this case, where F-actin was concentrated on the cell periphery while vimentin was accumulated in the perinuclear region. Cytoskeletal changes exposed to microgravity are also observed in muscular cell, immune cell, and stem cells, as reviewed recently [114]. Taken together, microgravity may be involved in YAP/TAZ mechanotransduction through cytoskeleton remodeling.

Cytoskeleton interacts with nucleus via the linker of nucleoskeleton and cytoskeleton complex. Actomyosin-generated cytoskeletal tension regulates nuclear stability and morphology [115]. For example, actin stress fibers formed in a stiff environment resulted in flattened nucleus with high aspect ratio in the vertical section in hepatic cells [116]. Cytoskeleton contraction-mediated deformation of nuclear envelope might stretch and widen nuclear pore complexes, which are a cylinder-like channel formed by the fusion of the outer and inner nuclear membranes, thereby enabling the nuclear-cytoplasmic shuttle of key molecules to influence gene expressions [117]. Force-induced opening of nuclear pore complexes in compressed nucleus reduced the mechanical resistance of molecular transport and permitted YAP nuclear import [118]. Similar nuclear accumulation of YAP/TAZ was observed in the compressed nucleus without intact cytoskeleton [119], suggesting that nuclear deformation directly regulated YAP/TAZ activation. In our previous studies, the impact of vector-directional gravity on nucleus translocation were studied for Ros 17/2.8 cells, MC3T3-E1 cells, and MSCs grown on upward-, downward- or edge-on-oriented substrates [120,121,122]. Nucleus longitudinal translocation presented a high value in downward orientation at 24 h and in edge-on orientation at 72 h, which was consistent with the distribution of perinuclear actin stress fibers and vimentin cords. This orientation-dependent nuclear translocation was also regulated by rearranged focal adhesion complex. Combined with a theoretical biomechanical model developed in our lab, it was proposed that gravity vector-directed nuclear translocation was mechanically balanced by the remodeled cytoskeleton and the reorganized focal adhesion complex [123], both of which were assumed to cause nuclear deformation reasonably in response to varied gravity vector orientation. Interestingly, after exposure to real microgravity, osteoblasts presented extended cell shapes as well as a significantly higher amount of disrupted and often fragmented or condensed nuclei [111,124]. Thus, microgravity-induced nuclear deformation may affect mechanically-mediated YAP/YAZ activation.

### 4.2. Phase Separation and YAP/TAZ Pathway

Phase separation is a well-known thermodynamic phenomenon in polymer chemistry, wherein a homogenous solution of molecules spontaneously separates into two coexisting phases: one enriched in (dense phase) and the other depleted of (dilute phase) these molecules [125,126]. Presented in the cells, biomolecules undergo liquid-liquid phase separation (LLPS), forming liquid-like droplets called membraneless organelles. Biomolecules in the dense phase are highly dynamic and able to freely exchange with their surroundings due to the absence of physical separation by the membrane [127]. Therefore, phase separation is sensitive to the changes in endogenous or exogenous factors including stress response, signal transduction, and posttranslational modifications [128].

Recently, both YAP and TAZ have been reported to form phase-separated condensates, as a novel manner, to promote transcriptional activity by engaging in super-enhancers [129]. Intrinsically-disordered transcription activation domain enabled YAP to undergo LLPS into cytoplasmic and nuclear condensates in response to hyperosmotic stress [130]. Nuclear YAP condensates recruited their co-activators TAZ and transcription factor TEAD to form super-enhancers that subsequently induced transcription of YAP target genes. After anti-PD-1 therapy in tumor cells, interferon-γ released from T cells promoted phase separation of YAP driven by hydrophobic interactions of the coiled-coil domain and weak interactions of the intrinsically disordered region in the C terminus [131]. YAP was phase partitioned with TEAD4, histone acetyltransferase EP300, and RNA polymerase recruiter mediator subunit 1 (Med1) to form nuclear condensates which were transcriptional hubs for maximizing target gene transcriptions of CD155 with resistance to immunotherapy. TAZ formed droplets in the nucleus through LLPS and this ability was negatively regulated by Hippo pathway in a coiled-coil domain-dependent manner [132]. TAZ nuclear condensates recruited TEAD4, coactivators bromodomain-containing protein 4, Med1, and transcription elongation factor CDK9 to initiate transcriptional activation. LLPS is also uncovered in other regulators in Hippo-YAP/TAZ pathway. In breast cancer cells, long noncoding RNA of small nucleolar RNA host gene 9 facilitated phase-separated droplets formation of LATS1 by interacting with its C-terminal domain [133]. LATS1 phase separation lost the capacity to sequester YAP in the cytoplasm and thus promoted YAP activation and cancer progression. Similarly, LATS1 was phase separated together with discoidin domain receptor 1 in response to stiff substrate or collagen binding, resulting in YAP translocation into nucleus freely [134]. In mouse embryonic stem cells, the hyper-activation of YAP after knockout of Mst1 and Mst2 induced the enrichment of Nanog, Sox2, Oct4 and H3 lysine 27 acetylation to form new super-enhancers and fostered phase-separated Med1 for driving ectoderm lineage differentiation [135].

Taken together, phase separation is illustrated to play important roles in regulating YAP/TAZ pathway. It is not clear whether microgravity could directly induce phase separation of YAP/TAZ or its regulators and thereby affect liver metabolism. At present, few studies focus on the relationship between phase separation and (micro-)gravity. Droplet-based microfluidics experiment showed that the growth dynamics of phase separated condensates reconstructed in vitro were dominated by gravity-induced coalescence [136]. In the microgravity-phase period of a parabolic flight, the phase separation of lysozyme, a selected model protein, was slower than under normal gravity, because the dynamics were driven by pure diffusion without convection inflight [137]. This conclusion was drawn based on the influence of gravity on the spinodal temperature of protein solution, in which the temperature was controlled by a controller with Peltier elements and measured with a Pt 100 sensor. Notably, temperature control and the related measurement are hard to control precisely due to a lack of convection. Furthermore, limited by the experimental techniques, only a time window of less than 20 s was allowed, preventing the observed effects of the microgravity environment on protein phase separation from pathological changes during spaceflight. To clearly present the effects of microgravity on phase separation of YAP/TAZ, it would be better to accomplish cell fixation and preservation in space, and visualize condensates formation by the immunofluorescence method, using a super-resolution microscope after returning to Earth. The appropriate cell lines, reliable experimental protocols, and suitable imaging methods all present challenges to carrying out such tests. Due to the limited positive results in the literature, whether the changes in the self-assembly dynamics of phase separation condensates regulate liver function through the YAP/TAZ pathway remains to be elucidated.

## 5. Conclusive Remarks and Perspectives

**Figure 3:**
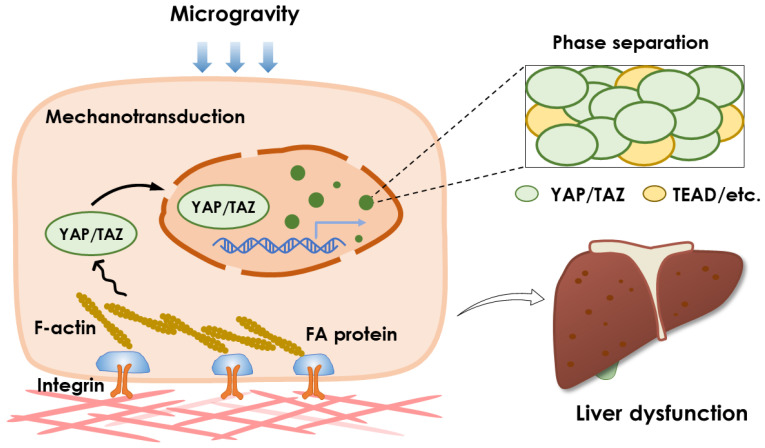
Mechanotransductive pathway and phase separation of YAP/TAZ may be responsible for liver dysfunctions induced by microgravity. Microgravity sensed by cells leads to cytoskeleton rearrangement and nuclear deformation, affecting YAP/TAZ cytoplasmic-nuclear distribution. Furthermore, YAP/TAZ undergoes LLPS and forms super enhancers with TEAD, Med1, and other molecules to promote transcription. This process is reasonably regulated by microgravity exposure since the weightless status delays phase separation of the modeled protein. Considering that YAP/TAZ is essential for liver metabolism, it is proposed that microgravity-dysregulated liver functions may result from YAP/TAZ inactivation through the disruption in mechanotransduction via cytoskeleton or nucleus, or disturbed phase separation via diffusion limitation.

Existing evidence indicates that liver function is dysregulated by spaceflight, mainly exhibiting the downregulated glycolysis, higher gluconeogenesis, and excess lipid storage. Unfortunately, the underlying molecular mechanism remains unclear. From the viewpoint of liver mechanobiology, YAP/TAZ tends to initiate various transcriptional activities upon perceiving extracellular mechanical stimuli, including microgravity, and its inactivation is potentiated to induce those abnormal liver metabolisms under microgravity coincidentally. Considering that microgravity can regulate YAP/TAZ activation, it is put forward here that liver dysfunctions during spaceflight might be derived from adaptive downregulation of YAP/TAZ signaling to microgravity stimuli (Figure 3).

One critical issue remains concerning whether gravitational force exerted on a cell is too small to induce any responses. Fortunately, growing evidence has demonstrated that cultured cells are sensitive to gravity [138,139]. Moreover, the specific structures or organelles discovered in cells, such as the statoliths in plants and the otoliths in the inner ear, are reported to have the capacity to sense gravitational signals [140]. In mammalian cells, although none of these specific structures are defined, the cytoskeleton and nucleus, the key components in mechanotransduction, could behave as mechanical sensors to adapt to gravity changes as described above. Furthermore, phase separation of YAP/TAZ emerged as a new manner to activate transcription. Microgravity exposure could even slow down the phase separation of signaling molecules due to reduced mass transfer in the diffusion-dominant environment [137]. Taken together, microgravity may regulate liver metabolism via YAP/TAZ mechanotransduction, while phase separation in the Hippo-YAP/TAZ pathway might disturb this process. Notably, (i) the link between YAP/TAZ and space phenotypes in the liver and (ii) the potential roles of mechanotransduction and phase separation in microgravity-regulated YAP/TAZ activation are both speculative proposals at this point. Readers are encouraged to critically assess the literature summarized here and carefully design experiments in future studies.

## Data Availability

No new data were created or analyzed in this study. Data sharing is not applicable to this article.
